# Clinical outcomes and prognostic factors in patients directly transferred to the intensive care unit from long-term care beds in institutions and hospitals: a retrospective clinical study

**DOI:** 10.1186/s12877-018-0950-9

**Published:** 2018-10-26

**Authors:** Su Hwan Lee, Soo Jung Kim, Yoon Hee Choi, Jin Hwa Lee, Jung Hyun Chang, Yon Ju Ryu

**Affiliations:** 1 Division of Pulmonary and Critical Care Medicine, Department of Internal Medicine, College of Medicine, Ewha Womans University, Mokdong Hospital, 1071 Anyangcheon-ro, Yangcheon-gu, Seoul, 07985 South Korea; 2 Department of Emergency Medicine, College of Medicine, Ewha Womans University, Mokdong Hospital, 1071 Anyangcheon-ro, Yangcheon-gu, Seoul, 07985 South Korea

**Keywords:** Long-term care, Nursing homes, Intensive care units, Pneumonia

## Abstract

**Background:**

There has been a steady increase in the aging population and an increase in the need for long-term care beds in institutions and hospitals (LTCHs) in Korea. The aim of this study was to investigate prognosis and to identify factors contributing to mortality of critically ill patients with respiratory problems who were directly transferred to intensive care units (ICU) from LTCHs.

**Methods:**

Following a retrospective review of clinical data and radiographic findings between July 2009 and September 2016, we included 111 patients with respiratory problems who had visited the emergency room (ER) transferred from LTCHs due to respiratory symptoms and who were then admitted to the ICU.

**Results:**

The mean age of the 111 patients was 79 years, and 71 patients (64%) were male. Pneumonia developed in 98 patients (88.3%), pulmonary thromboembolism in 4 (3.6%) and pulmonary tuberculosis in 3 (2.7%). Overall mortality was 19.8% (22/111). Multiple-drug-resistant (MDR) pathogens (odds ratio [OR], 17.43; 95% confidence interval [CI], 1.96–155.40) and serum albumin levels < 2.15 g/dL, which were derived through ROC (sensitivity, 72.7%; specificity, 85.4%) (OR, 28.05; 95% CI, 5.47–143.75), were independent predictors for mortality. The need for invasive ventilation (OR, 2.74; 95% CI, 1.02–7.32) and history of antibiotic use within the 3 months (OR, 3.23; 95% CI, 1.32–7.90) were risk factors for harboring MDR pathogens.

**Conclusions:**

The presence of MDR pathogens and having low serum albumin levels may be poor prognostic factors in patients with respiratory problems who are admitted to the ICU from LTCHs. A history of antibiotic use within the 3 months and the need for invasive ventilation can be helpful in choosing the appropriate antibiotics to combat MDR pathogens at the time of admission.

## Background

According to a recent statistical report, the number of people aged ≥65 is predicted to increase more from approximately 500 million in 2010 to approximately 1.5 billion in 2050, and the number of people aged ≥65 is expected to surpass that of those under 5 years old by 2050 [1]. As the proportion of the elderly population increases, the prevalence of various chronic diseases increases concomitantly, and the percentage of individuals considered frail also increases [2].

In Korea, the average life span is increasing and the proportion of elderly people aged ≥65 thus continues to rise. Combined with decreasing in fertility rates, Korea is becoming an aging society similar to the rest of the world [3]. The prevalence of chronic diseases and various age-related conditions including Alzheimer disease and stroke have increased. Accordingly, the need for long-term care beds in institutions and hospitals (LTCHs), defined as nursing and residential care facilities providing accommodations and long-term care as a package, is growing for this frail population [4–8].

Elderly patients living in LTCHs have a high incidence of chronic degenerative diseases [9–11] and frequently develop urinary tract infections and pneumonia [12–15]. Furthermore, elderly patients frequently experience unplanned transfers to general hospitals for various reasons [16]. These transfers generally result in patients being evaluated or managed by an emergency department that is likely to hospitalize patients [16], and some patients may be admitted to the intensive care unit (ICU) for treatment.

In Korea, a study of patients who visited the emergency department from LTCHs was reported previously [17]. However, studies on patients who were admitted to ICUs via emergency departments from LTCHs have not been conducted. Hence, we performed this study to investigate the factors associated with mortality in patients with respiratory problems who were directly transferred to the ICU from LTCHs.

## Methods

### Study design and subjects

This single-center study was a retrospective clinical study of all patients with respiratory problems who had been transferred from LTCHs to the ICU via the Emergency Department of Ewha Womans University Mokdong Hospital, which is a referral hospital with a 759-bed capacity containing 55 ICU beds (cardiovascular ICU, 7 beds; medical ICU, 15 beds; neuro ICU 18 beds; surgical ICU 15 beds) in Korea between July 2009 and September 2016. During this period, a total of 211 patients who had been transferred to our emergency department from LTCHs were hospitalized in the Pulmonary Department due to respiratory problems. Among these patients, 52.6% (111 of 211) required ICU admission.

### Data collection

Data from all patients admitted to the ICU were obtained through review the hospital’s electronic medical records. Variables for analysis were determined based on previous ICU mortality and LTCH studies [17–19]. Clinical data such as demographic characteristics, development of acute respiratory distress syndrome (ARDS), blood culture positivity, and mortality were evaluated. Various laboratory data such as albumin, C-reactive protein, and triglyceride levels were collected based on the first available data within 24 h after ICU admission. Sputum and bronchoalveolar lavage cultures were only collected within 48 h after admission to exclude infections after hospitalization. The severity of each patient’s condition was calculated according to the acute physiology and chronic health evaluation (APACHE) II score, and comorbidity was calculated using the Charlson comorbidity index [20, 21].

### Definition

A LTCH was defined as a medical institution with more than 30 beds for patients who need long-term hospitalization [7]. We excluded patients with LTCH stays of less than 3 days to exclude problems that occurred prior to LTCH admission.

The definition of a multi-resistant pathogen is based on non-susceptibility to at least one agent in three or more antimicrobial categories [22]. We considered several multiple-drug-resistant (MDR) pathogens including drug-resistant strains of *Pseudomonas aeruginosa*, carbapenem-resistant *Acinetobacter baumanii* (CRAB), *Stenotrophomonas maltophilia*, extended-spectrum β-lactamase (ESBL)-producing *Enterobacteriaceae* and methicillin-resistant *Staphylococcus aureus* (MRSA), all of which can be acquired during ICU hospitalization [23].

Acute kidney injury was defined as an increase in serum creatinine of ≥0.3 mg/dL within 48 h or an increase in serum creatinine to 30% more than the baseline level. Brain problems were defined as including neurodegenerative disorders, stroke, and brain hemorrhage.

### Statistical analysis

Demographic data are described as averages with standard deviation or numbers with percentage. For analysis between groups, data are described as medians with interquartile range or numbers with percentage. The Mann–Whitney U test or the chi-squared and Fisher’s exact tests were used to assess differences between groups. Logistic regression was used to investigate multivariate analysis for study of risk factors. Cut-off values for albumin were measured using receiver operating characteristic (ROC). The Kaplan–Meier method was used to report ICU length of stay and the survival curve, which were analyzed using the log-rank test. In all cases, a *p*-value of < 0.05 was considered statistically significant. SPSS version 23 (IBM, Armonk, NY, USA) was used for statistical analysis.

## Results

### Demographic characteristics of the overall study population

**Fig. 1:**
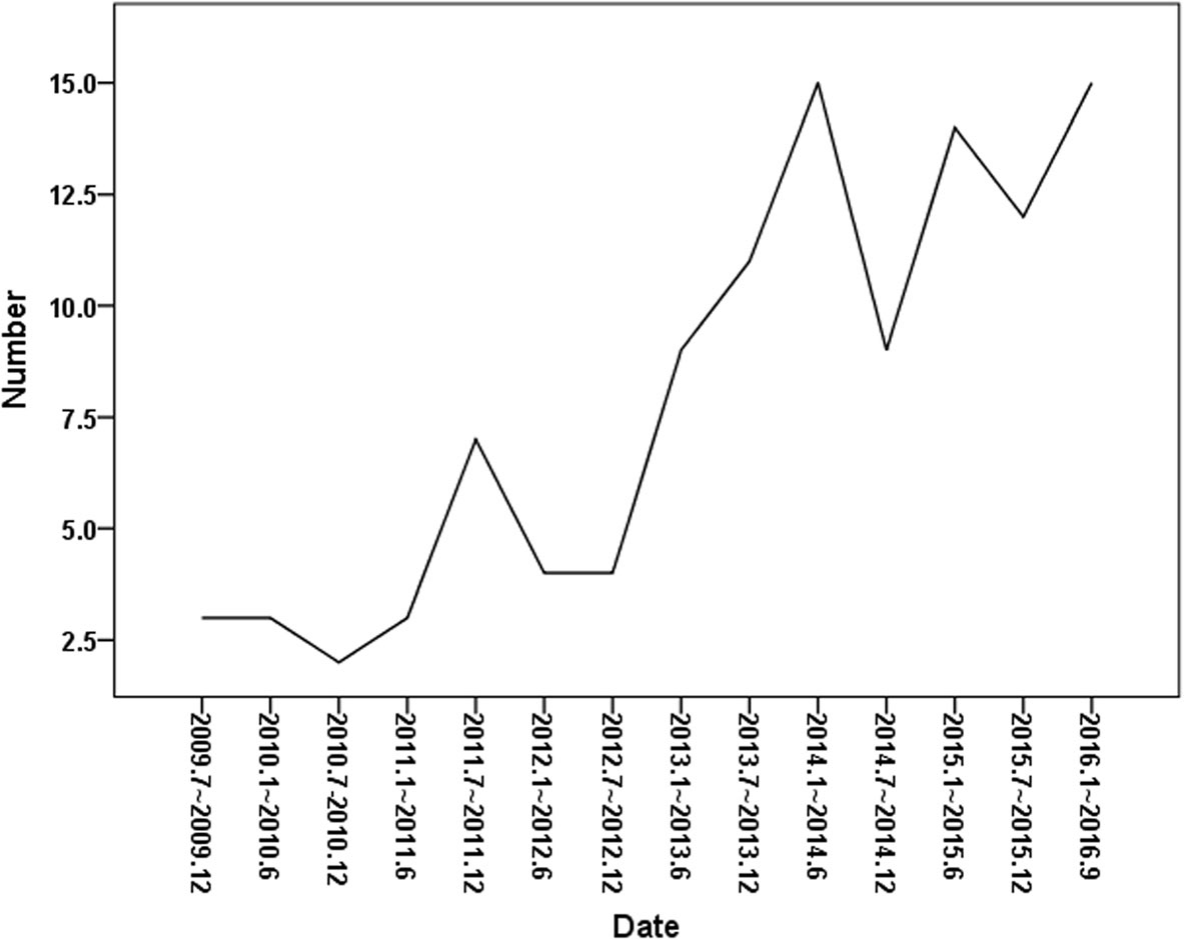
Trends in the number of patients with respiratory problems admitted to the ICU from long-term care beds in institutions and hospitals

The number of patients admitted to the ICU via the emergency department from LTCHs gradually increased during the study period (Fig. 1). The patient population comprised a higher proportion of males than females (64% vs. 36%) who were of advanced age (mean age, 79 years). Pneumonia (*n* = 98) was the leading cause requiring a visit to the hospital, followed by pulmonary thromboembolism (*n* = 4), active pulmonary tuberculosis (*n* = 3), idiopathic pulmonary fibrosis (*n* = 1), exacerbation of chronic obstructive pulmonary disease (*n* = 1), hemoptysis (*n* = 1), lung cancer (*n* = 1), and asphyxia (*n* = 1). Seventy-seven patients (69.4%) developed acute respiratory failure requiring invasive ventilation. The mean APACHE II score was 24.9, and the mean Charlson comorbidity index was 2.45. The incidence rates of ARDS and positive blood culture were 26.1% and 22.5%, respectively. Among the sputum cultures that were performed for patients within 48 h of admission, 66 patients had positive sputum cultures, and more than 2 pathogens were identified in 8 patients. The mean ICU length of stay was 11.8 days, and the mean hospital length of stay was 24.5 days. The 28-day mortality rate was 15.3% (17/111), and the all-cause mortality rate during hospitalization was 19.8% (22/111). Two of the surviving patients were discharged to their homes, while all other survivors were transferred to other LTCHs. Baseline characteristics were not significantly different from 2009 to 2016 except sputum MDR pathogen species. CRAB was not identified before 2012, but has been consistently identified since 2012. Other MDR pathogens were similarly identified during the study period. Table 1 shows the baseline characteristics of the study population.

**Table 1:** Demographic and clinical characteristics of the study population

Characteristics	*N* = 111
Male sex	71 (64)
Age, years	79 ± 10
BMI, kg/m^2^	20.7 ± 4.0
APACHE II score	24.9 ± 6.7
Charlson Comorbidity Index	2.45 ± 1.67
ICU length of stay, days	11.8 ± 1.3
Hospital length of stay, days	24.5 ± 3.0
Permanent tracheostomy status	28 (25.2)
Nasogastric tube insertion status	38 (34.2)
Bedridden status	50 (45)
ARDS	29 (26.1)
Use of antibiotics within 3 months	49 (44.1)
Blood culture positive	25 (22.5)
Sputum culture positive	66 (59.5)
MDR pathogen (sputum culture or blood culture)	53 (47.7)
Major cause of admission	
Pneumonia	98 (88.3)
Pulmonary thromboembolism	4 (3.6)
Pulmonary tuberculosis	3 (2.7)
Lung abscess	1 (0.9)
Idiopathic pulmonary fibrosis	1 (0.9)
Lung cancer	1 (0.9)
COPD exacerbation	1 (0.9)
Asphyxia	1 (0.9)
Hemoptysis	1 (0.9)

Values are presented as numbers (percentages) and as mean ± standard deviation, unless otherwise indicated. BMI, body mass index; APACHE, acute physiology and chronic health evaluation; ICU, intensive care unit; ARDS, acute respiratory distress syndrome; MDR, multiple-drug-resistant; COPD, Chronic Obstructive Pulmonary Disease

### Comparison of characteristics between survivors and non-survivors

Study populations were divided into survivor and non-survivor groups according to all-cause mortality. The results of this inter-group comparison are shown in Table 2. The variables of sex, median age, median severity score, tracheostomy or nasogastric tube insertion, use of antibiotics within 3 months, development of ARDS, and acute kidney injury did not significantly differ between the survivor and non-survivor groups. However, the non-survivor group had lower median body mass index (BMI) compared with the survivor group (18.3 vs. 20.8, *P* = 0.016). Identification of MDR pathogens was also significantly higher in the non-survivor group relative to the survivor group (86.4% vs. 38.2%, *P* < 0.001). Regarding the laboratory parameters that were routinely analyzed in ICU patients, serum albumin and fasting triglyceride levels both measured significantly low in the non-survivor group. In particular, the proportion of patients whose serum albumin levels were < 2.15 g/dL, which was derived through ROC (sensitivity, 72.7%; specificity, 85.4%), as compared with the proportion of patients with serum albumin levels ≥2.15 g/dL was higher in the non-survivor group than in the survivor group (*P* < 0.001).

**Table 2:** Predictors of mortality between survivors and non-survivors

Variable	Univariate			Multivariate	
	Survivor (*n* = 89)	Non-survivor (*n* = 22)	*P*	*OR (95% CI)*	*P*
Male sex	55 (61.8)	16 (72.7)	0.339	0.31 (0.06–1.75)	0.186
Age, years	78 (69.5–83.0)	81.5 (74.3–86.3)	0.064	1.07 (0.96–1.08)	0.143
BMI, kg/m^2^	20.8 (18.7–23.7)	18.3 (15.9–22.3)	0.016	1.05 (0.85–1.31)	0.643
APACHE II score	25.0 (21.0–29.5)	25.5 (22.0–30.3)	0.459		
Charlson Comorbidity Index	2 (1–3)	2 (1–3)	1.000		
Blood culture positive	21 (23.6)	4 (18.2)	0.777		
Permanent tracheostomy status	26 (29.2)	2 (9.1)	0.052	0.35 (0.04–3.02)	0.340
Nasogastric tube insertion status	30 (33.7)	8 (36.4)	0.814		
Invasive ventilation	58 (65.2)	19 (86.4)	0.053	4.02 (0.68–23.76)	0.124
ARDS	21 (23.6)	8 (36.4)	0.222		
Use of antibiotics within 3 months	37 (41.6)	12 (54.5)	0.273		
MDR pathogen	34 (38.2)	19 (86.4)	< 0.001	17.43 (1.96–155.40)	0.010
MRSA	8 (9)	10 (45.5)	< 0.001		
CRAB	16 (18)	7 (31.8)	0.238		
Bedridden status	44 (49.4)	6 (27.3)	0.061	0.24 (0.05–1.13)	0.071
Brain problem^a^	69 (77.5)	15 (68.2)	0.36		
Diabetes mellitus	20 (22.5)	7 (31.8)	0.36		
Hypertension	37 (41.6)	7 (31.8)	0.402		
Acute kidney injury	27 (30.3)	4 (18.2)	0.255		
Albumin, g/dL	2.6 (2.3–2.9)	2.0 (1.7–2.2)	< 0.001		
Albumin < 2.15 g/dL	13 (14.6)	16 (72.7)	< 0.001	28.05 (5.47–143.75)	< 0.001
Total bilirubin, mg/dL^b^	0.5 (0.3–0.8)	0.6 (0.4–0.7)	0.627		
LDH, IU/L	239 (185.5–307.5)	258 (200.5–367.0)	0.184		
CRP, mg/dL	13.0 (7.3–23.6)	16.3 (12.8–21.3)	0.151		
NT-ProBNP pg/mL	1337 (410–4462)	2909.5 (1302.5–10,555.3)	0.124		
Triglyceride, mg/dL^c^	76.0 (55.0–106.3)	54.0 (43.0–83.0)	0.034		
ICU length of stay	7 (3–14.5)	5.5 (3.5–19)	0.801		

Values are presented as numbers (percentages), and as median (interquartile range), unless otherwise indicated. OR, odds ratio; CI, confidence interval; BMI, body mass index; APACHE, acute physiology and chronic health evaluation; ARDS, acute respiratory distress syndrome; MDR, multiple-drug resistant; MRSA, methicillin-resistant *Staphylococcus aureus*; CRAB, carbapenem-resistant *Acinetobacter baumannii*; LDH, lactate dehydrogenase; CRP, C-reactive protein; NT-ProBNP, N-terminal prohormone of brain natriuretic peptide; ICU, intensive care unit; ^a^neurodegenerative disorders and stroke; ^b^Five missing values; ^c^Fourteen missing values

**Fig. 2:**
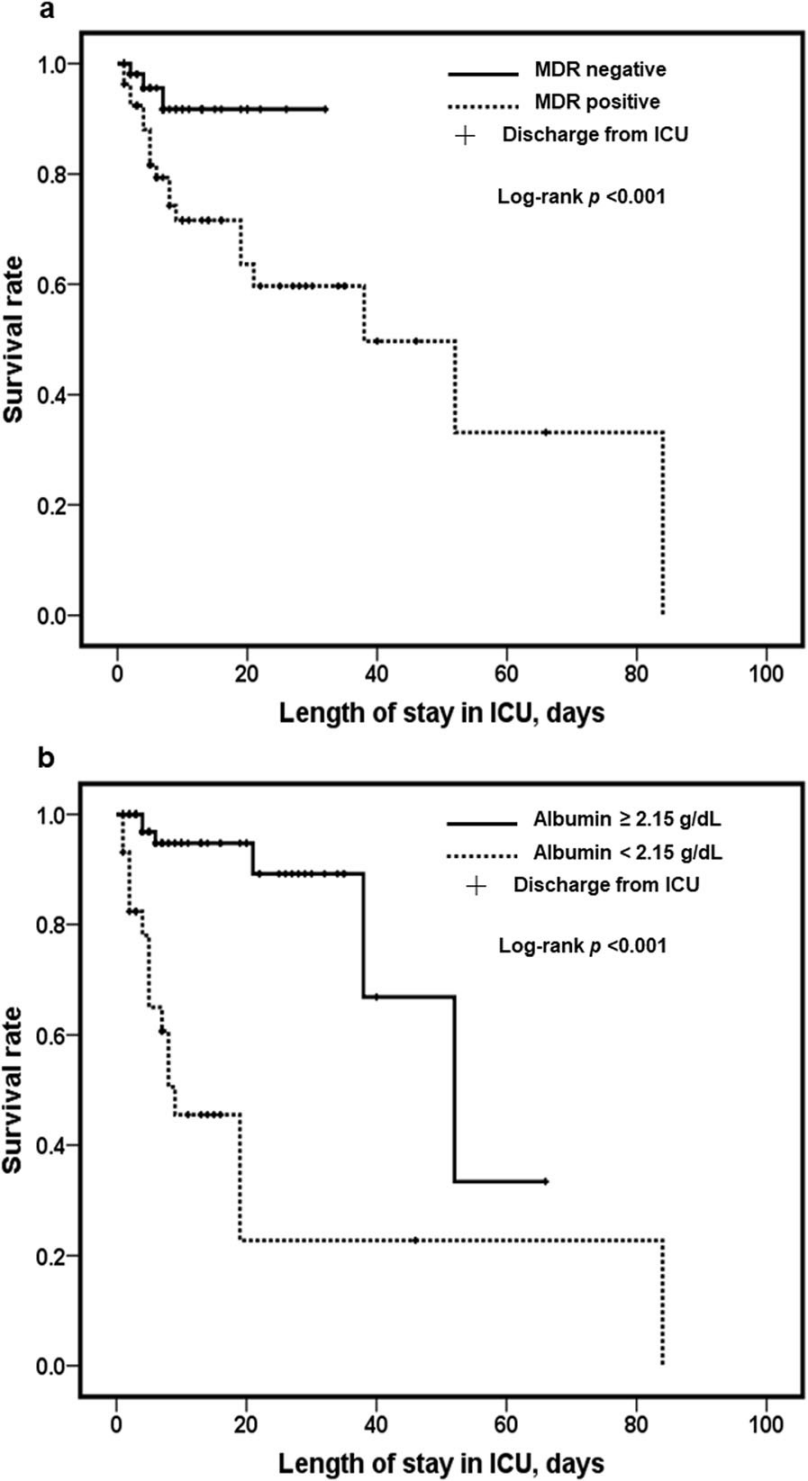
**a**, **b** Kaplan-Meier survival analysis of mortality and ICU length of stay according to identification of MDR pathogen and albumin levels

Multivariate analysis showed that the MDR pathogen identification (odds ratio [OR], 17.43; 95% confidence interval [CI], 1.96–155.40) and serum albumin levels < 2.15 g/dL (OR, 28.05; 95% CI, 5.47–143.75) were associated with increased mortality. Statistical analysis of groups divided according to MDR pathogen or albumin level showed significant differences in survival rate and ICU length of stay (Fig. 2).

### Risk factors for identification of MDR pathogens

Further analysis of MDR pathogens was performed, because the previous analysis demonstrated a difference between identification of MDR pathogens in the survivor and non-survivor groups. We identified 74 bacterial pathogens from the sputum cultures of 66 patients, and more than 2 bacterial pathogens were identified in 8 of the patients (Table 3). Among the 74 bacterial pathogens, 49 (66.2%) were MDR. The most common pathogens were CRAB (31.1%) followed by MRSA (23%).

**Table 3:** Distribution of isolated sputum culture pathogens within 48 h after admission

Pathogen identified	*N* = 74
Gram-positive pathogen	
*Staphylococcus aureus*	20 (27.0)
MSSA	2 (2.7)
MRSA	18 (24.3)
*Streptococcus pneumonia*	5 (6.8)
Gram-negative pathogen	
*Acinetobacter baumannii*	24 (32.4)
*Escherichia coli*	2 (2.7)
*Klebsiella pneumonia*	7 (9.5)
*Enterobacter cloacae*	1 (1.4)
*Stenotrophomonas maltophilia*	2 (2.7)
*Pseudomonas aeruginosa*	5 (6.8)
*Serratia marcescens*	1 (1.4)
*Proteus mirabilis*	4 (5.4)
*Mycobacterium tuberculosis*	3 (4.1)
MDR pathogen	49 (66.2)
MRSA	18 (24.3)
ESBL producing *Enterobacteriae*	7 (9.5)
MDR-*Pseudomonas* spp.	1 (1.4)
CRAB	23 (31.1)

Values are presented as numbers (percentages) unless otherwise indicated; MSSA, methicillin-susceptible *Staphylococcus aureus*; MRSA, methicillin-resistant *Staphylococcus aureus*; MDR, multiple-drug-resistant; ESBL, extended-spectrum beta-lactamases; CRAB, carbapenem-resistant *Acinetobacter baumannii*

In 25 patients with positive blood cultures, 12 MDR bacterial pathogens were identified including 3 MRSA, 3 methicillin-resistant coagulase-negative *Staphylococci* spp., 1 MDR *Streptococcus*, 2 CRAB and 3 ESBL-producing *Enterobacteriaceae* spp. There were 53 patients with MDR pathogens in sputum or blood cultures compared with 58 patients who did not have MDR pathogens.

A comparison of the MDR-negative and MDR-positive groups is shown in Table 4. The MDR-positive group had a higher proportion of males (77.4% vs. 51.7%, *P* = 0.005) and lower BMI (19.2 vs. 20.8, *P* = 0.005) than the MDR-negative group. At the time of admission, the presence of tracheostomy or nasogastric tube insertion did not differ between the two groups. The need for invasive ventilation, history of antibiotics use within the 3 months, and proportion of serum albumin level < 2.15 g/dL were higher in the MDR positive group. After multivariate analysis, the need for invasive ventilation (OR, 2.74; 95% CI, 1.02–7.32) and history of antibiotics use within the 3 months (OR, 3.23; 95% CI, 1.32–7.90) were shown to be risk factors for MDR pathogens.

**Table 4:** Risk factors for MDR pathogens in patients who transferred from long-term care beds in institutions and hospitals

Variable	Univariate			Multivariate	
	MDR negative (*n* = 58)	MDR positive (*n* = 53)	*P*	*OR (95% CI)*	*P*
Male sex	30 (51.7)	41 (77.4)	0.005	2.65 (1.02–6.93)	0.046
Age, years	78 (70.8–82)	79 (71–84)	0.372	1.02 (0.97–1.07)	0.373
BMI, kg/m^2^	20.8 (19.4–25.3)	19.2 (17.5–22.7)	0.005	0.9 (0.80–1.02)	0.102
Permanent tracheostomy status	17 (29.3)	11 (20.8)	0.300		
Nasogastric tube insertion status	16 (27.6)	22 (41.5)	0.123		
Invasive ventilation	34 (58.6)	43 (81.1)	0.010	2.74 (1.02–7.32)	0.045
Bedridden status	25 (43.1)	25 (47.2)	0.667		
Brain problem^a^	46 (79.3)	38 (71.7)	0.350		
Use of antibiotics within 3 months	16 (27.6)	33 (62.3)	< 0.001	3.23 (1.32–7.90)	0.010
Diabetes mellitus	16 (27.6)	11 (20.8)	0.402		
Hypertension	25 (43.1)	19 (35.8)	0.435		
Chronic pulmonary disease	7 (12.1)	13 (24.5)	0.088		
Acute kidney injury	19 (32.8)	12 (22.6)	0.235		
Albumin < 2.15 g/dL	8 (13.8)	21 (39.6)	0.002	2.85 (0.98–8.24)	0.054

Values are presented as numbers (percentages), and as median (interquartile rage), unless otherwise indicated; MDR, multiple-drug-resistant; OR, odds ratio; CI, confidence interval; BMI, body mass index; ^a^neurodegenerative disorders and stroke

## Discussion

In this study, we found that the number of patients with respiratory problems admitted to the ICU from LTCHs increased gradually over the study period, and those who harbored MDR pathogens or had low serum albumin levels at the time of admission showed lower survival rates. A history of antibiotic use within 3 months and the need for invasive ventilation were risk factors for MDR pathogen infection.

The major cause of hospitalization for respiratory problems was pneumonia. Infection with MDR pathogens is associated with a worse prognosis than infection with susceptible pathogens, and the rapid administration of appropriate antibiotics is critical for a good prognosis [24–26]. In our study, the presence of MDR pathogens, which plays an important role in the selection of appropriate antibiotics, was also associated with mortality in patients from LTCHs. Previous studies about community-acquired pneumonia or healthcare-associated pneumonia reported that home infusion therapy, immunosuppression, chronic kidney disease, tube feeding, aspiration, LTCHs, a history of prior hospitalization, and a history of antibiotic use within the 3 months were risk factor for MDR pathogen infection [27–30]. Because our study subjects mostly had chronic diseases and brain problems or were frail, we supposed that only a history of antibiotics and the need for invasive ventilation were shown as risk factors. Many subjects in our study already had nasogastric tubes, tracheostomies, or were bedridden, all of which have been shown to be risk factors for developing MDR infections [28, 30]. The need for invasive ventilation indicates poor lung condition. Some patients transferred from our emergency department to LTCHs were already using antibiotics. Thus, the need for invasive ventilation may be a result of non-response to current antibiotic treatment, which may suggest the possibility of MDR pathogen infection.

Lastly, our study showed that male sex was associated with infection with MDR pathogens. In our study, males were not older than females, and there were no significant differences in ARDS, nasogastric tube, BMI, or other variables. Males showed higher a proportion of antibiotic use within 3 months than females; however, this difference was not statistically significant. It is possible that cigarette smoking have affected the results [31], because Korean males have a higher prevalence of smoking than Korean females [32]. A study reported male sex to be a risk factor for MDR pathogen infection similar to our results [28]. Unfortunately, we could not clarify the relevance because it was difficult to definitively document smoking history due to cognitive issues in many patients.

Serum albumin levels are a strong predictor of all-cause mortality in acutely admitted medical patients [19, 33, 34]. Our study also showed that hypoalbuminemia was correlated with mortality. As previous studies demonstrated that hypoalbuminemia is often associated with malnutrition and malnutrition is associated with mortality [19, 35], we initially thought that the cause of the association of hypoalbuminemia and mortality was the nutritional status of the patients. Furthermore, univariate analysis demonstrated that the non-survivor group had a lower median BMI compared to the survivor group. However, other studies have reported that albumin is not a good marker of malnutrition for elderly patients [36, 37]; rather, they suggest that the decrease in albumin, which acts as a binding protein, increases the circulation of unbound drugs, which may lead to adverse events [33]. In addition, serum albumin can decrease dramatically in critical illness, which may cause problems such as transport of substances, anti-thrombotic effects, and maintenance of normal plasma oncotic pressure. In elderly patients, increases in the bioavailability of medications due to impaired protein homeostasis and taking various medications might explain adverse events [38]. In our study, most patients from LTCHs were elderly; only 16 patients were under 65 years of age. We supposed that these several reasons induced serum albumin level was associated with mortality in patients who transferred from LTCHs. Furthermore, APACHE II score and Charlson comorbidity index did not vary between groups in this study. Albumin levels might be a better marker for severity or prediction of length of stay in ICU in elderly patients from LTCHs.

This study had some limitations. First, since this study was performed at a single center, it is difficult to generalize the trend to LTCHs throughout Korea. However, to the best of our knowledge, there are no other studies investigating patients with respiratory problems who were transferred to ICU from LTCHs in Korea. Therefore, this study may have important implications for aging societies such as Korea and other similar countries. Second, this was a retrospective study, thus we could only use data included in electronic medical records. The subjects’ status and medication history at the time of admission were documented in records from LTCH doctors; however, some data were difficult to confirm due to patient cognitive difficulties, including the infectious history of subjects, the drug sensitivity of pathogens during LTCH hospitalization, and survey data including smoking history and degree of symptoms. Third, we could not exclude colonization or contamination by the identified pathogens; thus, such pathogens may be included in our analysis. To overcome these challenges, more prospective studies are needed.

## Conclusion

Identification of MDR pathogens and low serum albumin levels were associated with poor prognosis in patients with respiratory problems who were admitted to the ICU from LTCHs. A history of antibiotic use within the 3 months and the need for invasive ventilation were associated with infection with MDR pathogens; thus, these factors should be considered when selecting appropriate antibiotics at admission. Additionally, serum albumin levels at admission may be helpful for predicting prognosis.

## Abbreviations

APACHE: Acute physiology and chronic health evaluation
ARDS: Acute respiratory distress syndrome
BMI: Body mass index
CI: Confidence interval
ESBL: Extended-spectrum β-lactamase
HCAP: Healthcare-associated pneumonia
ICH: Intensive care unit
LTCHs: Long-term care beds in institutions and hospitals
MDR: Multiple-drug-resistant
MRSA: Methicillin-resistant Staphylococcus aureus
OR: Odds ratio
ROC: Receiver operating characteristic
